# Transcriptional activation of *USP16* gene expression by NFκB signaling

**DOI:** 10.1186/s13041-019-0535-3

**Published:** 2019-12-30

**Authors:** Shou Yang, Juelu Wang, Shipeng Guo, Daochao Huang, Isabel Bestard Lorigados, Xing Nie, Dandan Lou, Yanhua Li, Mingjing Liu, Yu Kang, Weihui Zhou, Weihong Song

**Affiliations:** 1 Chongqing City Key Lab of Translational Medical Research in Cognitive Development and Learning and Memory Disorders, and Ministry of Education Key Lab of Child Development and Disorders, Children’s Hospital of Chongqing Medical University, Chongqing, 400014 China; 2 Townsend Family Laboratories, Department of Psychiatry, The University of British Columbia, 2255 Wesbrook Mall, Vancouver, BC V6T 1Z3 Canada

**Keywords:** Down syndrome, *USP16*, NFκB, Transcriptional regulation, Promotor

## Abstract

Ubiquitin Specific Peptidase 16 (*USP16*) has been reported to contribute to somatic stem-cell defects in Down syndrome. However, how this gene being regulated is largely unknown. To study the mechanism underlying *USP16* gene expression, *USP16* gene promoter was cloned and analyzed by luciferase assay. We identified that the 5′ flanking region (− 1856 bp ~ + 468 bp) of the human *USP16* gene contained the functional promotor to control its transcription. Three bona fide NFκB binding sites were found in *USP16* promoter. We showed that p65 overexpression enhanced endogenous *USP16* mRNA level. Furthermore, LPS and TNFα, strong activators of the NFκB pathway, upregulated the *USP16* transcription. Our data demonstrate that *USP16* gene expression is tightly regulated at transcription level. NFκB signaling regulates the human *USP16* gene expression through three *cis*-acting elements. The results provide novel insights into a potential role of dysregulation of *USP16* expression in Alzheimer’s dementia in Down Syndrome.

## Introduction

Down syndrome (DS) is a complex developmental disorder caused by genetic defects, leading to intellectual and developmental disabilities. It is a result of complete or partial trisomy of chromosome 21 [1, 2]. Individuals with DS therefore have three copies of 161 known protein-encoding genes. The phenotypes of DS are believed to be related with abnormal gene expression and functions due to the extra copy of the genes on chromosome 21 [3, 4], and the DS patients invariably develop Alzheimer’s disease (AD)-related neuropathology [5–10]. Although a few candidate genes have been linked to the spectrum of disorders associated with DS [11–16], it is unclear that how trisomy of specific genes contributes to the disease.

The human *USP16* gene is mapped on chromosome 21 and triplicated in DS. *USP16* gene contains 3 mRNA transcripts which share the same start codon, stop codon, and translation frame. *USP16* has been reported to contribute to the somatic stem-cell defects in DS and reduce the self-renewal of multiple somatic stem cells [17], suggesting that some of the pathological features associated with DS may result from a stem-cell imbalance due to overexpression of *USP16*. It was first identified as a histone H2A specific deubiquitinase that regulates cell cycle progression and gene expression in human cells [18]. This deubiquitinating enzyme, *USP16*, removes the ubiquitin protein from H2A-K119, and upregulates the transcription of the Ink4a locus [17]. The Ink4a locus encodes the p16^Ink4a^ and the p19^Arf^ genes, which are important members participating in self-renewal and senescence pathways. It was reported that *USP16* was upregulated in response to DNA damage, and the upregulation of its expression was HECT and RCC1-like domain-containing protein 2 (HERC2)- dependent [19]. Furthermore, *USP16* was shown to regulate embryonic stem cell gene expression and hematopoietic stem cell function [20, 21]. A recent study reported that *USP16* was involved in cancer, and its downregulation promoted hepatocellular carcinoma cells growth [22]. The converging lines of evidence shed light on *USP16* ‘s functions, but the transcriptional regulation of *USP16* gene is largely unknown.

NFκB signaling pathway plays an important role in the gene regulation [23–25] and is associated with inflammation [26], oxidative stress [27], and apoptosis [28]. The mammalian NFκB family consists of five members, including NFκB1 (p50), NFκB2 (p52), RelA (p65), RelB, and C-Rel [29]. These members form various homo- or heterodimeric complexes. Activation of NFκB is tightly controlled by an inhibitory subunit, known as the inhibitor of NFκB (IκB). IκB binds to NFκB dimers to block their nuclear localization sequences, thus NFκB dimers are retained within the cytoplasm [30]. Once cells are stimulated by activators, such as tumor necrosis factor-α (TNFα) and lipopolysaccharide (LPS) [31], IκB is phosphorylated by IκB kinase (IKK) complex, making itself being degraded by ubiquitin-proteasome pathway [32]. Then NFκB dimers are released and translocated into the nucleus, where they regulate the transcription of the NFκB target genes [29].

Previous studies have shown that NFκB plays essential roles in cell cycle progression [33], senescence [34], DNA damage repair [35], maintenance of stem cells pluripotency [36] and cancer. In the present study, we aim to elucidate how *USP16* gene expression is regulated and the role of NFκB in *USP16* gene regulation. We cloned and functionally analyzed the human *USP16* gene promoter region. We showed that the *USP16* gene promoter contained functional *cis*-acting NFκB binding sites. By using EMSA, we identified three bona fide binding sites, through which NFκB signaling regulates *USP16* gene transcription. p65 overexpression was shown to increase the endogenous *USP16* mRNA level and the activators of the NFκB pathway, including LPS and TNFα, also upregulated the *USP16* transcription. By knocking out p65 in mice embryonic fibroblasts, the effects of TNFα on upregulating *USP16* transcription was abolished.

## Materials and methods

### Primers and plasmids construction

The 5′ flanking region of the human *USP16* gene was amplified by polymerase chain reaction (PCR) from human genomic DNA. The primers were designed with restriction enzymes sites compatible with multi-cloning sites of vector pGL4.10 (Promega). The pGL4.10 vector lacks eukaryotic promoter and enhancer sequences upstream of a reporter luciferase gene. We first cloned the longest 2324 bp (− 1856 bp ~ + 468 bp) promotor region into pGL4.10 at the *Xho*I and *Hind*III sites to generate p*USP16*-A. Then, promotor deletion assays were conducted as previously described [25, 37]. Briefly, a series of deletion fragments were amplified by using p*USP16*-A as the template and sub-cloned into pGL4.10 at proper restriction enzymes sites. All used primers were listed in Additional file 1.

### Cell culture, luciferase assays, and transfection

Human embryonic kidney 293 (HEK293) (RRID:CVCL_0045) and human neuroblastoma SH-SY5Y cell lines (RRID:CVCL_0019) were cultured in Dulbecco’s modified Eagle’s medium (DMEM, Gibco) containing 10% fetal bovine serum (FBS, Gibco). Wildtype (WT) mouse embryonic fibroblasts (MEFs) and p65 knockout (KO) MEFs were maintained in DMEM supplemented with 15% FBS, β-mercaptoethanol, and ESGRO (LIF) [24]. All cells were maintained in a 37 °C incubator containing 5% CO_2_. For luciferase assays, pCMV-Rluc (Promega) was co-transfected with p*USP16-*related promoter plasmids as a control to normalize the transfection efficiency. Specifically, 270 ng p*USP16-*related promoter plasmids and 30 ng pCMV-Rluc were cotransfected into each well of a 48 well-plate by using 0.9 μl Lipofectamine-™2000 reagent (Invitrogen). Cells were harvested 24 h after transfection and lysed with 60 μl 1 × passive lysis buffer (Promega) per well. Activities of the *Firefly* and *Renilla* luciferases from the same sample were sequentially assayed by a luminometer (GloMax 20/20) following the protocol of the dual-luciferase reporter assay system (Promega, E1910). The *Firefly* luciferase activity was normalized by the *Renilla* luciferase activity and the results reflected the relative promoter activity. For RNA extraction analysis, 4 μg plasmid DNA was transfected by 12 μl Lipofectamine-™2000 reagent per well of a 6 well-plate.

### Electrophoretic mobility shift assay (EMSA)

EMSA was performed as previously described [38]. To obtain NFκB-enriched nuclear extract, HEK293 cells were transfected with the p65 expression plasmid (pMTF-p65) for 24 h. Nuclear protein was extracted by using NE-PER™ nuclear and cytoplasmic extraction reagents (Thermo Scientific) according to the manufacturer’s instructions. Five oligonucleotides probes were labeled with IR700 dye (Bioneer Corporation) and annealed with corresponding anti-sense oligonucleotides to generate double-stranded probes at a final concentration of 0.01 pmol/μl. Among them, *USP16* 3x NFκB contained three NFκB *cis*-acting elements, including NFκB2, NFκB3, and NFκB4. *USP16*-NFκB1, *USP16*-NFκB2, *USP16*-NFκB3, and *USP16*-NFκB4 oligonucleotide had a corresponding sequence. For competition experiments, 2 μl of nuclear extract was incubated with 0.01 pmol/μl of labeled probes and 100× (1 pmol/μl) unlabeled competition probes for 20 min at room temperature. For the supershift assay, monoclonal anti-NFκB p65 antibody (Cell Signaling, 8424 s) was added. The reaction mixtures were separated on a 4% Tris-glycine-EDTA gel for 70 min at 70 V in darkness. The gel was scanned using LI-COR Odyssey (LI-COR Biosciences) at a wavelength of 700 nm. The sequences of the oligonucleotides were listed in Additional file 1.

### LPS and TNFα treatment

LPS (Sigma, L4516) was reconstituted in DMEM and further diluted to a final concentration of 50 ng/ml, while TNFα (Sigma, H8916) was reconstituted in sterile phosphate buffered saline (PBS) containing 0.1% endotoxin-free recombinant human serum albumin at a final concentration of 10 ng/ml. For qRT-PCR, HEK293 and SH-SY5Y cells were exposed to LPS and TNFα at the proper concentration for 24 h and then lysed for RNA extraction. For RT-PCR, MEF WT and p65 KO cells were treated with 5 ng/ml TNFα for 24 h.

### qRT-PCR

Total RNA was extracted from cells using TRI reagent (BioTeke, RP1202), and quantified with Nanodrop 2000 (Thermo Fisher Scientific, Waltham, MA, USA). PrimeScript™ RT reagent Kit (Takara, RR037A) was used to synthesize the first-strand cDNA from an equal amount of various RNA samples according to the manufacturer’s instructions. qRT-PCR was performed by using SYBR® Premix Ex Taq™ II (Takara, RR820A) and the PCR program included one initial denaturation step at 95 °C for 3 min, 39 cycles of 95 °C for 10 s, 58 °C for 30s, and 72 °C for 30 s (Bio-Rad CFX96). Glyceraldehyde 3-phosphate dehydrogenase (GAPDH) was used as an endogenous control. Primers used in this assay were listed in Additional file 1. Amplification efficiency of those primers were checked by standard curve method, with an E value around 100% (R^2^ > 0.998). The relative expression of mRNA was calculated with the 2^-ΔΔT^method. Each sample was triplicated. qRT-PCR data were analyzed and converted to relative fold changes. Additionally, total RNA was extracted from MEF WT or p65 KO cells by TRIzol reagent (Invitrogen). Thermoscript™ SuperScript IV first-strand synthesis system (Invitrogen) was applied to amplify the first-strand cDNA by using 1.5 μg of total RNA as the template and then the newly synthesized cDNA was used as the template to perform PCR by Taq DNA polymerase. A pair of primers to amplify a 150 bp region of mice *USP16* gene was as follows: forward, 5′- ctgccaagactgtaagactgac, and reverse, 5′- ggtgtcgtgtagtgcttcaag. Additionally, a pair of primers for amplifying a 205 bp fragment of mouse GAPDH gene coding sequence was as follows: forward, 5′- ggatttggtcgtattggg, and reverse, 5′- ggaagatggtgatgggatt. All samples were analyzed on 2.5% agarose gels.

### Statistical analyses

Three or more independent experiments were performed. All results were presented as mean ± the standard error of the mean (SEM) and 2-tailed Student’s *t* test was used to analyze the difference between two groups. One-way Analysis of variance (ANOVA) was applied to analyze the data in Fig. 2 and multiple comparison tests were conducted by *post-hoc* Turkey’s method. Statistical analysis in Fig. 3 and Fig. 5e-f was performed by two-way ANOVA followed by *post-hoc* Turkey’s multiple comparisons test. *p* < 0.05 was considered as statistically significant.

## Results

### Cloning of the human *USP16* promoter

**Fig. 1:**
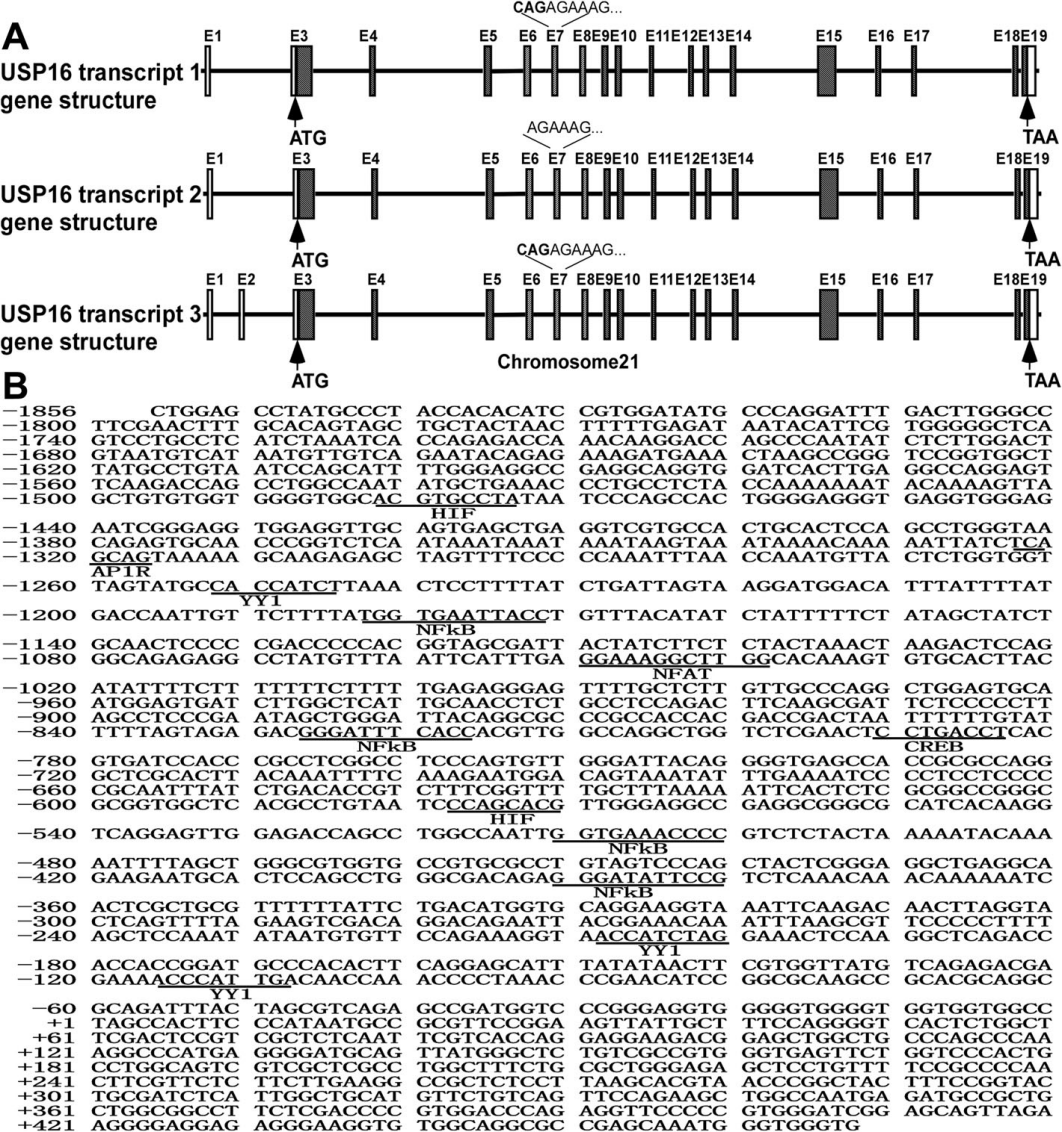
Sequence of the human *USP16* gene promotor. **a** The genomic structure of the human *USP16* gene on Chromosome 21. E stands for exon. *USP16* consists of 19 exons. ATG is the translation start codon located in E3 and TAA is the stop codon. **b** The nucleotide sequence of the human *USP16* gene promotor. A 2324 bp fragment of the 5′ flanking region of the human *USP16* gene was cloned from HEK293 genomic DNA. The Thymine + 1 represents the first base of transcription in E1. The putative transcription factor binding sites are underlined in bold

The human *USP16* gene is mapped on chromosome 21, at 21q21.3. It has three mRNA transcripts which share the same start codon, stop codon, and translation frame. Transcript 1 and 3 encode the same isoform a and transcript 2 encodes isoform b. Transcripts 3 is the longest one which contains all the 19 exons, while the other two lack Exon 2. The only difference between transcript 1 and transcript 2 is that the later is shorter and lacks the first codon “CAG” in Exon 7 (Fig. 1a). Human genomic DNA were extracted from HEK293 cells and a 2324 bp 5′ flanking region of the *USP16* gene was amplified by PCR. The human *USP16* gene has a complex transcriptional machinery as suggested by the results from a computer-based transcription factor binding site search using Genomatix and TFSearch. The human *USP16* gene promoter was shown to contain several putative regulatory elements, such as MAF and AP1 related factors (AP1R), NFκB, hypoxia-inducible factor (HIF), nuclear factor of activated T-cells (NFAT), cAMP-responsive element binding proteins (CREB), and Ying Yang 1 (YY1) (Fig. 1b). There are 4 putative NFκB binding sites spanning a wide region of USP16 gene promoter in our predication results and NFκB signaling pathway is an essential player in the inflammatory response, which has been recognized as a factor to facilitate the pathogenesis of DS and AD phenotypes in DS patients [39]. Therefore, we mainly focused on NFκB signaling pathway in the following experiments.

### Functional analysis of the human *USP16* gene promoter

**Fig. 2:**
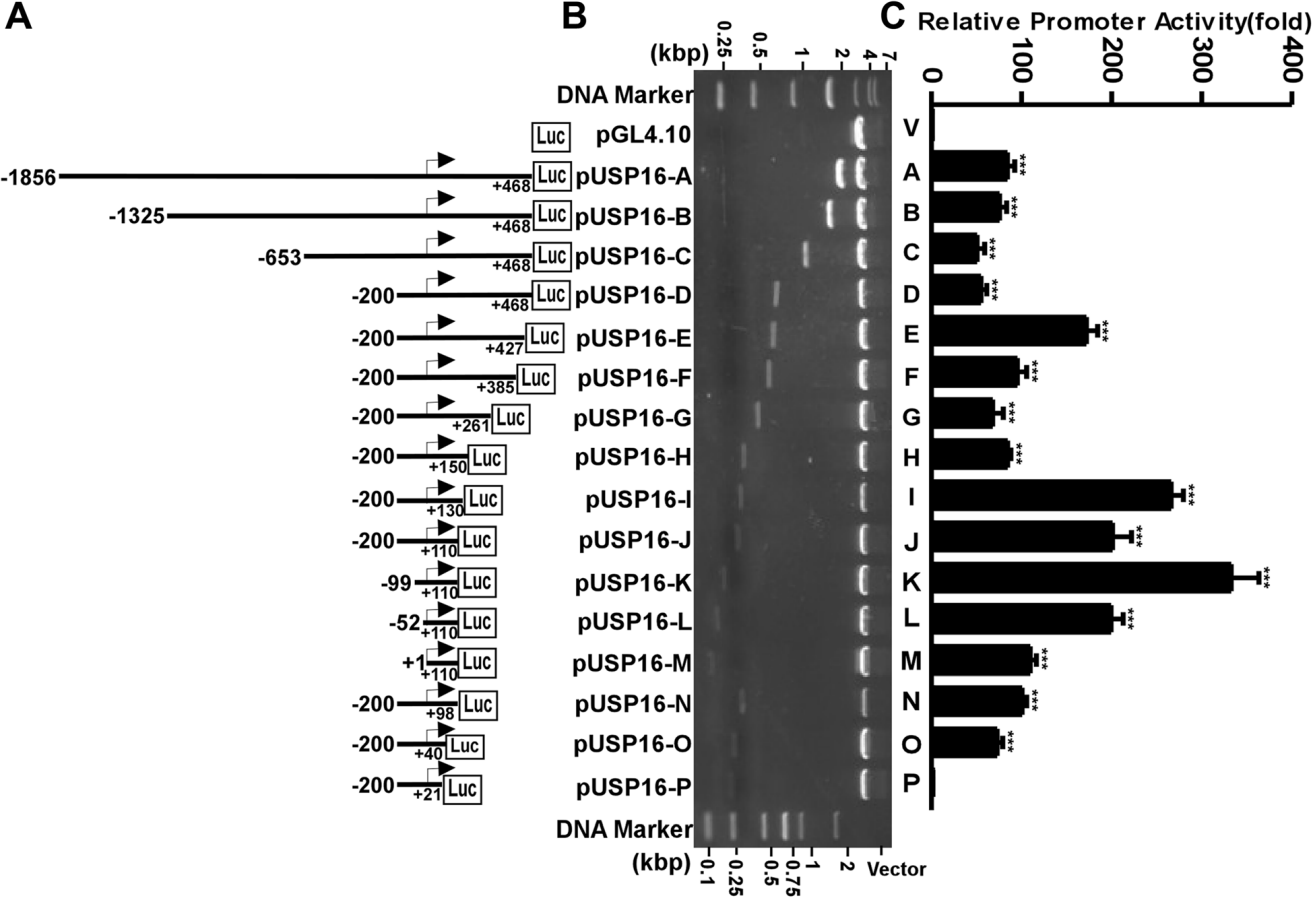
Deletion analysis of the human *USP16* gene promoter. **a** Schematic diagram of the human *USP16* deletion promoter constructs in pGL4.10 vector. Arrow shows the direction of transcription. The numbers represent the start and end points for each construct. **b** The deletion plasmids were confirmed by restriction enzyme digestion, and the digested samples were analyzed on a 1.0% agarose gel. The vector size is 4.2 kb; *USP16* promoter fragment size ranges from 0.11 to 2.3 kb. The sequences of the inserts were further confirmed by sequencing. **c** The promoter plasmids were co-transfected with pCMV-Luc into HEK293 cells. After 24 h transfection, the cells were harvested and luciferase activity was measured with a luminometer and presented in relative luciferase units (RLU). The pCMV-Luc luciferase activity was used to normalize for transfection efficiency. The values represent means ± SEM. *n* = 3, ***p < 0.001 by one-way ANOVA test followed by *post-hoc* Turkey’s test. Comparisons were made between all *USP16* promoter reporter plasmids and the empty pGL4.10 as a negative control

To determine the functional promoter region of the human *USP16* gene, we cloned a 2324 bp 5′ flanking region of the *USP16* gene into a promoter-lacking vector pGL4.10 to generate p*USP16*-A (− 1856 bp to + 468 bp) plasmid. The luciferase activities of cells transfected with this plasmid largely rely on the presence of a functional promoter upstream of a luciferase gene. The plasmid p*USP16*-A was transfected into HEK293 cells, and luciferase activity was measured by a GloMax 20/20 Luminometer to examine its promoter activity. Compared with cells transfected with an empty vector pGL4.10, p*USP16*-A showed a significant increment of luciferase activity (84.48 ± 4.55RLU, *p* < 0.0001) (Fig. 2c). This result indicates that the 2324 bp fragment contains the functional promoter region of the human *USP16* gene.

To identify the regulatory elements in the *USP16* promotor region, a series of deletion fragments within p*USP16*-A was generated (Fig. 2a). The luciferase assays of these deletion plasmids were performed. The results indicated that p*USP16*-B (− 1325 bp~ + 468 bp, 75.91 ± 4.40RLU; *p* > 0.9999), p*USP16*-C (− 653 bp~ + 468 bp, 50.98 ± 4.74RLU; *p* = 0.1065), and p*USP16*-D (− 200 bp~ + 468 bp, 55.21 ± 3.57 RLU; *p* = 0.2514) have no significant changes of luciferase activities when compared with p*USP16*-A (− 1856 bp to + 468 bp, 84.48 ± 4.55RLU). A series of 3’end deletion plasmids was constructed and transfected into HEK293 cells. Luciferase activities of these plasmids showed that a 41 bp deletion (p*USP16*-E, − 200 bp ~ + 427 bp, 172.13 ± 7.16RLU) from p*USP16*-D (− 200 bp~ + 468 bp, 55.21 ± 3.57RLU) greatly increased promotor activity (*p* < 0.0001), indicating that there are negative regulatory elements located in this region. A further 42 bp deletion (p*USP16*-F, − 200 bp ~ + 385 bp, 95.36 ± 5.98RLU) significantly reduced promotor activity when compared to p*USP16*-E (− 200 bp ~ + 427 bp, 172.13 ± 7.16RLU) (*p* < 0.0001), while deletion plasmids p*USP16*-G (− 200 bp ~ + 261 bp, 68.06 ± 6.89RLU; *p* = 03525) and p*USP16*-H (− 200 bp ~ + 150 bp, 84.89 ± 1.88RLU; *p* = 0.9995) did not show significant changes to that of p*USP16*-F (− 200 bp ~ + 385 bp, 95.36 ± 5.98RLU). However, a further 20 bp deletion (p*USP16*-I, − 200 bp ~ + 130 bp, 266.32 ± 7.80RLU) from p*USP16*-H (− 200 bp ~ + 150 bp) largely enhanced promotor activity (*p* < 0.0001), indicating that the 20 bp region contains negative regulatory elements.

Further deletion analysis found that a 20 bp deletion from p*USP16*-I (− 200 bp~ + 130 bp, 266.32 ± 7.80RLU) to p*USP16*-J (− 200 bp~ + 110 bp, 201.22 ± 12.10RLU, *p* < 0.0001) and a 12 bp deletion from p*USP16*-J (− 200 bp~ + 110 bp) to p*USP16*-N (− 200 bp~ + 98 bp, 100.74 ± 2.94RLU, *p* < 0.0001) substantially reduced promotor activity, while a 48 bp deletion from p*USP16*-N (− 200 bp~ + 98 bp) to p*USP16*-O (− 200 bp~ + 40 bp, 73.13 ± 3.51RLU) did not significantly change the promoter activity (*p* = 0.3357). However, a further 19 bp deletion from p*USP16*-O (− 200 bp~ + 40 bp) to p*USP16*-P (− 200 bp~ + 21 bp, 1.74 ± 0.07RLU) almost abolished promotor activity (*p* < 0.0001). These results showed that + 40 bp was the proper 3′ boundary of the *USP16* promotor region. Since we found p*USP16*-J (− 200 bp ~ + 110 bp, 201.22 ± 12.10RLU) still had a high promotor activity compared with the empty vector pGL4.10 (*p* < 0.0001), p*USP16*-J was chosen to investigate the effect of 5’end on promotor activity. A series of 5’end deletion plasmids was constructed and transfected into HEK293 cells. Luciferase activity showed that a 101 bp deletion (p*USP16*-K, − 99 bp~ + 110 bp, 332.87 ± 17.59RLU) from p*USP16*-J (− 200 bp~ + 110 bp) increased *USP16* promotor activity (*p* < 0.0001), while a 47 bp deletion (p*USP16*-L, − 52 bp~ + 110 bp, 199.65 ± 7.69RLU) from p*USP16*-K (− 99 bp~ + 110 bp) decreased *USP16* promotor activity (*p* < 0.0001) and a further 52 bp deletion (p*USP16*-M, + 1 bp~ + 110 bp, 109.72 ± 3.88RLU) from p*USP16*-L (− 52 bp~ + 110 bp) reduced *USP16* promotor activity (*p* < 0.0001). Taken together, our data illustrate that the promoter region from + 1 bp to + 40 bp has the minimal promoter activity required for basal transcription and various *cis*-acting regulatory elements are located in the 5′ flanking region of *USP16* gene.

### NFκB upregulates the human *USP16* gene promotor activities

**Fig. 3:**
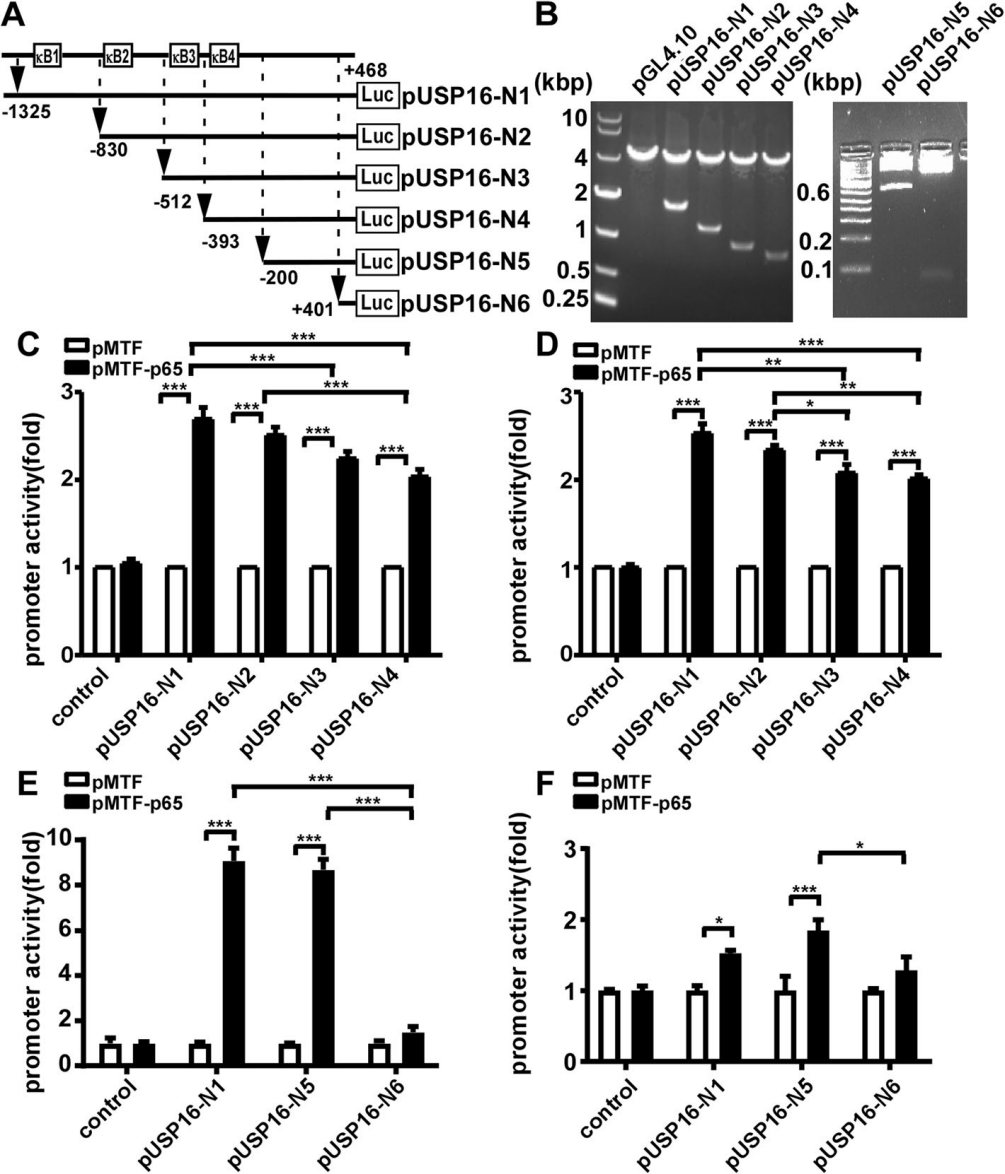
*USP16* promoter activity is up-regulated by NFκB p65. (**a**) Schematic diagram of deletion plasmids containing different human *USP16* promoter fragments in front of the *firefly* luciferase reporter gene of the pGL4.10 vector. (**b**) The deletion plasmids corresponding to p*USP16-*N1, −N2, −N3, −N4, −N5 and –N6 were checked by sequencing and restriction enzyme digestion and the digested samples were analyzed on 1% or 3.5% agarose gels. Vector size is 4.2 kb and the *USP16* promoter fragment insert size ranges from 69 bp to 2.2kbp. *USP16* promoter deletion plasmids and pMTF-p65 or pMTF were co-transfected into (**c, e**) HEK293 cells and (**d, f)** SH-SY5Y cells with pGL4.10 as a control. After transfection for 24 h, the cells were harvested and luciferase activity was presented in relative luciferase units (RLU). The pCMV-Luc luciferase activity was used to normalize for transfection efficiency. The values represent means ± SEM. n = 3, * *p* < 0.05, ** *p* < 0.01, ***p < 0.001 by two-way ANOVA followed by *post-hoc* Turkey’s multiple comparisons test

Computer-based transcription factor binding site analysis revealed four putative NFκB *cis*-acting elements in the 1793 bp (− 1325 bp ~ + 468 bp) promoter region of the human *USP16* gene (Fig. 1b). To determine whether NFκB signaling regulates *USP16* gene transcription by interacting with these putative NFκB *cis*-acting elements, the effects of NFκB overexpression on the promoter activity of the 1793 bp region were examined. Four human *USP16* promoter deletion constructs, p*USP16*-N1, −N2, −N3, and -N4, were cloned into pGL4.10 vector, with sequential elimination of one upstream putative NFκB-binding element (Fig. 3a, b). We used a dual-luciferase reporter assay to examine the *USP16* promoter activity in HEK293 cells overexpressed with NFκB p65 expression plasmid (pMTF-p65) or empty vector (pMTF). Compared with empty vector pMTF, NFκB p65 expression plasmid co-transfecting with p*USP16*-N1, p*USP16*-N2, p*USP16*-N3, and p*USP16*-N4 resulted in enhanced luciferase activities to about 2.69, 2.51, 2.24, and 2.04 folds, respectively (*p* < 0.0001) (Fig. 3c). Furthermore, if comparing the luciferase activities among p*USP16-*N1, −N2, −N3, and N4 plasmids after NFκB p65 overexpression, the statistical significance was observed between p*USP16-*N1 vs. p*USP16-*N3 (*p* = 0.0009), p*USP16-*N1 vs. p*USP16-*N4 (*p* < 0.0001) and p*USP16-*N2 vs. p*USP16-*N4 (*p* = 0.0004). These suggested that deletion of binding site 2 and 3 had substantial impact on NFκB p65’s role in upregulating *USP16* gene promoter. In SH-SY5Y cells, similar results were observed for NFκB p65’s effects on increasing the activities of *USP16* gene promoter (*p* < 0.0001) (Fig. 3d). To further confirm the involvement of p65 in regulating *USP16* gene promoter, two more deletion constructs, p*USP16*-N5 and -N6, were generated. Both p*USP16*-N5 and -N6 plasmids do not contain any putative NFκB-binding element (Fig. 3a, b). Unexpectedly, the promoter activity of p*USP16-*N5 was still significantly increased to 8.67 folds in HEK cells (*p* < 0.0001) and to 1.85 folds in SH-SY5Y cells (*p* = 0.0005) by p65 overexpression compared with the empty vector transfection. A further deletion fragment, p*USP16-*N6, was completely abolished the effect of p65 overexpression on affecting its promoter activity (Fig. 3e, f), suggesting a non-canonical NFκB binding site sequence located in p*USP16*-N5 was indirectly affected by p65 [40]. Taken together, these results demonstrate that NFκB p65 up-regulates human *USP16* promoter activity.

### *USP16* promoter activity is regulated through three NFκB binding elements

**Fig. 4:**
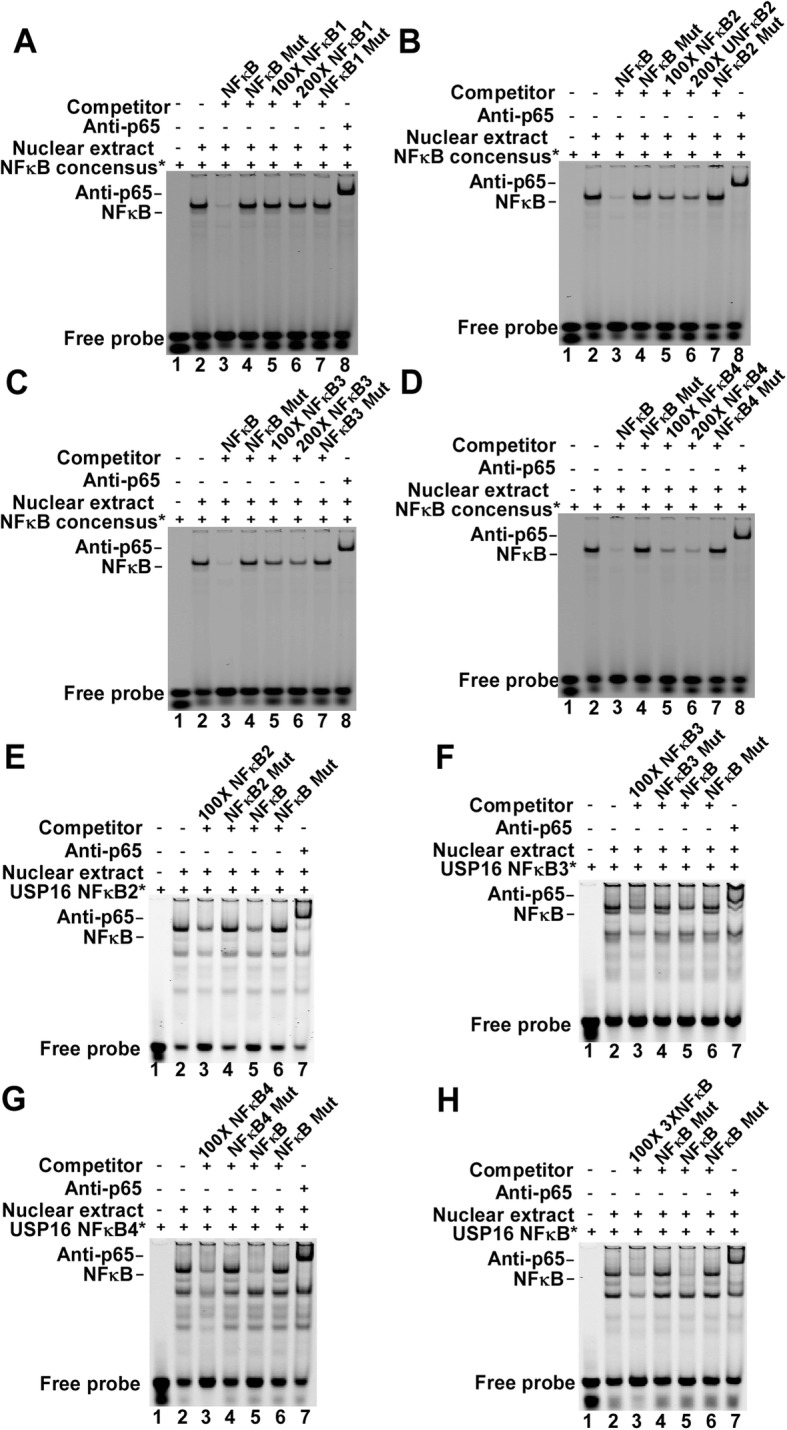
Gel mobility shift assay for the *USP16* gene promoter. Gel shift and Super gel shift assays were performed as described in the Materials and methods. **a-d** Double-stranded consensus NFκB oligonucleotides were end-labeled with IR700 dye as probes. Incubation of labeled probe with nuclear extracts formed a shifted DNA-protein complex band (lane 2). For competition assays, different concentrations of unlabeled competition oligonucleotides, consensus NFκB (lanes 3), mutant NFκB (lanes 4), *USP16* NFκB (lanes 5 and lanes 6), *USP16* NFκB mutant (lanes 7) were added. Anti-NFκB p65 antibody was used for the super gel shift assay. Addition of the anti-NFκB p65 antibody into the reaction mixture produced a supershifted band, indicating the formation of the nuclear protein-*USP16*-p65 complex (lane 8). **e-g** *USP16* NFκB2, *USP16* NFκB3, and *USP16* NFκB4 double-stranded oligonucleotides were end-labeled with IR700 dye as probes, respectively. Incubation of labeled probe with nuclear extracts formed a shifted DNA-protein complex band (lane 2). For competition assays, unlabeled competition oligonucleotides, *USP16* NFκB (lanes 3), *USP16* NFκB mutant (lanes 4), consensus NFκB (lanes 5), mutant NFκB (lanes 6) were added. Anti-NFκB p65 antibody was used for the super gel shift assay. The anti-NFκB p65 antibody supershifted the nuclear protein-*USP16*-p65 complex (lane 7). **h** A double-stranded oligonucleotide contains *USP16* NFκB2, *USP16* NFκB3, and *USP16* NFκB4 cis-elements were end-labeled with IR700 dye as probe for EMSA

To investigate which putative NFκB binding elements of *USP16* interact with NFκB p65, we performed EMSA to determine whether four putative NFκB *cis*-acting elements physically bind to NFκB p65. We synthesized NFκB consensus oligonucleotides end-labeled with IR700 dye as probes and four oligonucleotides (*USP16* NFκB 1–4) containing each NFκB *cis*-acting element as competitors. Labelled NFκB consensus probes were visualized as a heavy band on the bottom of the gel (Fig. 4b, lane 1), and a shift band was formed after the addition of the p65-enriched nuclear extracts (Fig. 4b, lane 2), suggesting DNA- protein complex formation. Moreover, the shift band was abolished when NFκB consensus, 100 × *USP16* NFκB2, or 200 × *USP16* NFκB2 (Fig. 4b; lanes 3, 5, 6) was applied, but not for NFκB mutant or *USP16* NFκB2 mutant (Fig. 4b; lanes 4, 7). Addition of anti-NFκB p65 antibody resulted in a slower migrating super shifted band (Fig. 4b, lane 8), confirming the existence of NFκB in the complex formation. Similar results were seen in *USP16* NFκB3 group (Fig. 4c) and *USP16* NFκB4 group (Fig. 4d), but not in *USP16* NFκB1 group (Fig. 4a). The data suggests that the second, third and the fourth NFκB-binding elements in *USP16* gene are able to interact with NFκB p65.

To further confirm these results, we performed additional EMSA with the three NFκB *cis*-acting elements (*USP16* NFκB2,3,4) end-labeled with IR700 dye as probes. Each probe contained a single putative NFκB *cis*-acting site. A shifted protein-DNA complex band was detected after incubating *USP16* NFκB2 probe with NFκB-enriched nuclear extract (Fig. 4e, lane 2). This shifted band was abolished by addition of 100× *USP16* NFκB2 or NFκB consensus oligonucleotide (Fig. 4e; lanes 3, 5), but not *USP16* NFκB2 mutant or NFκB mutant (Fig. 4e; lanes 4, 6). Super EMSA was performed to further confirm the existence of NFκB elements in the *USP16* NFκB2 probe. Addition of anti-NFκB p65 antibody resulted in a slower migrating super shifted band (Fig. 4e, lane 7), Similar results were also obtained for *USP16* NFκB3 (Fig. 4f), *USP16* NFκB4 (Fig. 4g). In addition, we synthesized a single probe (*USP16* 3x NFκB) containing three NFκB *cis*-acting elements and gained similar results (Fig. 4h). The data demonstrated that the second, third, and the fourth NFκB binding elements in *USP16* gene interact with NFκB p65.

### Activation of NFκB signaling increases the human *USP16* gene transcription

**Fig. 5:**
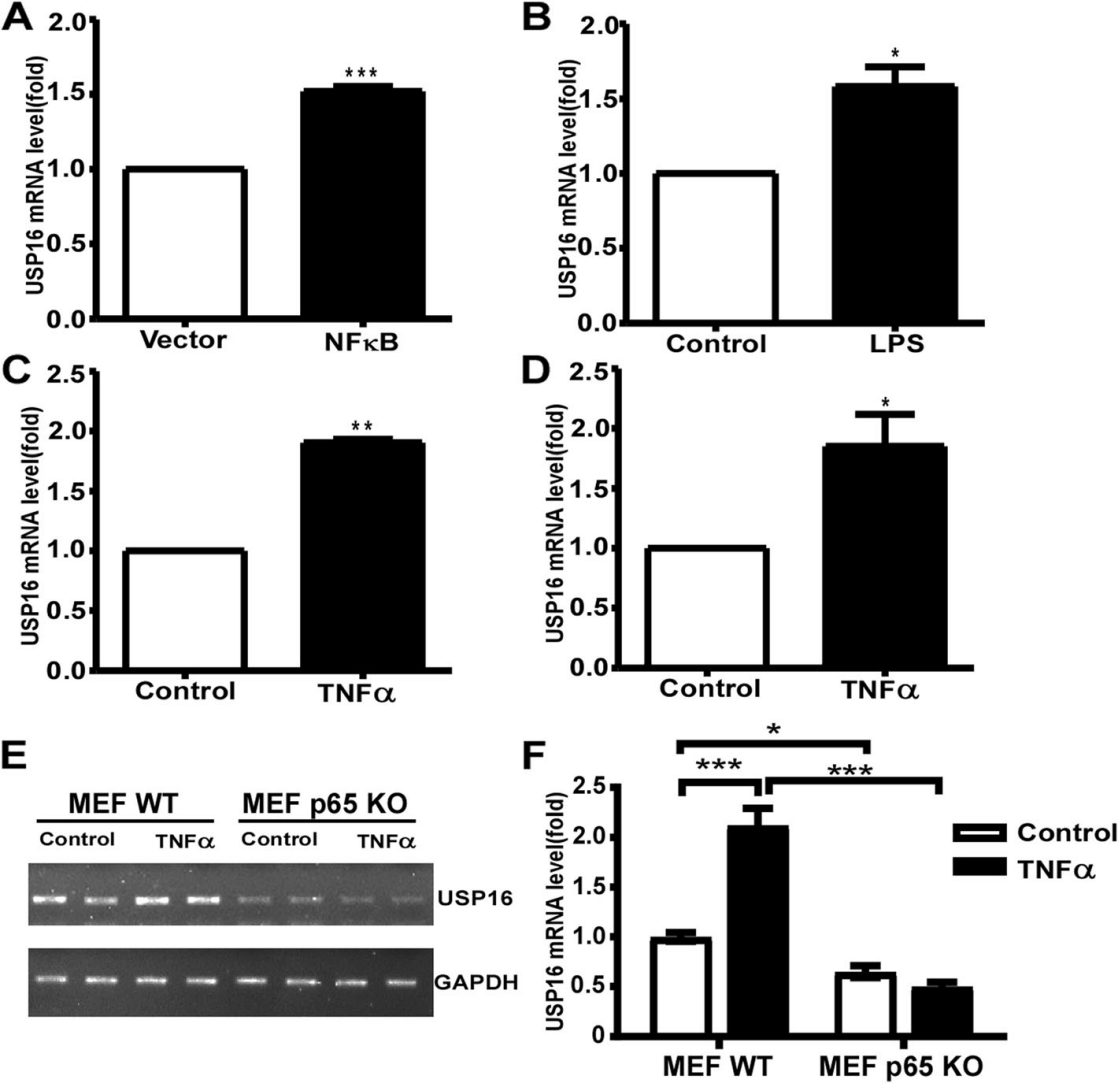
Enhancement of *USP16* transcription in response to p65, LPS and TNFα. (**a**) HEK293 Cells were transfected with either empty vector (pMTF) or the p65 expression plasmids (pMTF-p65) for 24 h, and *USP16* mRNA levels were determined by qRT-PCR. (**b**) SH-SY5Y cells were exposed to LPS at 50 ng/ml for 24 h. Total RNA was extracted. The mRNA levels of *USP16* were determined by qRT-PCR and normalized against the levels of GAPDH. HEK293 cells (**c**) and SH-SY5Y cells (**d**) were exposed to TNFα at 10 ng/ml for 24 h. The mRNA levels of endogenous *USP16* gene were determined by qRT-PCR and normalized against the levels of GAPDH. All data are presented as mean ± SEM. n = 3, *p < 0.01, by analysis of variance with Student’s t-test. MEF WT and p65 KO cells (**e**) were exposed to TNFα at 5 ng/ml for 24 h, and mRNA levels of *USP16* and GAPDH were examined by RT-PCR. (**f**) The endogenous mRNA levels of *USP16* were normalized against the levels of GAPDH and analyzed by two-way ANOVA test followed by *post-hoc* Turkey’s test. The values represent means ± SEM. n = 3, ***p < 0.001

To examine whether NFκB affects the endogenous gene transcription, qRT-PCR was conducted to measure the endogenous *USP16* mRNA level. Overexpression of NFκB markedly upregulated endogenous mRNA level of the *USP16* gene compared to the control group (1.52-fold, *p* < 0.0001) (Fig. 5a). LPS and TNFα are strong activators of NFκB signaling pathway. To explore whether NFκB binding elements in the human *USP16* promoter mediate the inflammatory effect on *USP16* transcription, we examined *USP16* mRNA levels after LPS and TNFα treatment. HEK293 cells or SH-SY5Y cells were treated with LPS (50 ng/ml) or TNFα (10 ng/ml) for 24 h. Similar to the effect of NFκB overexpression on endogenous *USP16* mRNA levels (Fig. 5a), stimulation of LPS resulted in a marked increase of endogenous *USP16* mRNA levels in SH-SY5Y cells (1.55-fold, *p* = 0.0364) (Fig. 5b), but no significant effect in HEK293 cells (data not shown). However, TNFα enhanced the levels of endogenous *USP16* mRNA both in HEK293 cells (1.90-fold, *p* = 0.0011) (Fig. 5c) and SH-SY5Y cells (1.85-fold, *p* = 0.0327) (Fig. 5d). In order to confirm p65 directly mediating the effect of TNFα on upregulating *USP16* transcription, TNFα was further applied to a p65 knockout fibroblast cell line (MEF) and *USP16* mRNA levels were examined. As shown in Fig. 5e and f, endogenous *USP16* mRNA levels was significantly decreased to 0.65 folds by knocking out p65 in MEF cells (*p* = 0.0397), suggesting p65 is a strong activator for endogenous *USP16* gene expression. Furthermore, TNFα treatment significantly increased *USP16* mRNA levels by 1.12 folds compared with control (*p* < 0.0001), whereas such increasement was abolished in MEF p65 KO cells. These data suggest that activation of NFκB signaling pathway by p65, LPS and TNFα upregulates *USP16* gene transcription. Taken together, endogenous *USP16* gene transcription was enhanced by activators of the NFκB pathway, including LPS and TNFα, and p65 knockout abolished the effects of TNFα on upregulating *USP16* gene transcription.

## Discussion

*USP16* protein is a histone H2A-specific deubiquitinase with the coding gene located on human chromosome 21. Several previous studies suggest that it plays many roles in gene expression, cell cycle progression, cell self-renewal, and senescence pathways [17, 18]. *USP16* affects hematopoiesis and hematopoietic stem cell function [20]. In mouse hematopoietic stem cells (HSCs), knock out of *USP16* was associated with a reduction of mature and progenitor cell populations, while HSC number did not change. *USP16* was reported to be involved in hepatocellular carcinoma and decreased expression of *USP16* by carboxyl-terminal truncated HBx (Ct-HBx) in live tumor cells promoted stem-like properties [22]. By using DS model mice (Ts65Dn), triplication of *USP16* contributed to neuronal progenitor defects and abnormal development of mammary epithelium. Overexpression of *USP16* in human fibroblast cultures manifested growth impairment and senescence, reminiscent of DS’s cells [17]. Additionally, *USP16* was shown to interact with HERC2 to regulate ubiquitin signaling during DNA repair during DNA damage response. This could be essentially important to DS, since cellular response to DNA damage was altered in DS patient cells [19].

In this study, we demonstrated that NFκB enhanced human *USP16* gene transcription. We first identified that the 5′ flanking region of *USP16* gene, from -1856 bp to + 468 bp, showed promotor activity. Then we found several functional regulatory elements by a series of promotor deletion analysis. By using a computer-based analysis, we determined four putative NFκB binding sites in the promotor region. After conducting EMSA, we provided solid evidence to support that three binding sites (*USP16* NFκB2, *USP16* NFκB3, and *USP16* NFκB4) physically interacts with p65. In this article, we first proved that p65 overexpression could enhance endogenous *USP16* mRNA levels through three *cis*-acting elements. It was unexpected that the promoter activity of the p*USP16*-N5 plasmid without any putative p65 binding site was still increased by p65 overexpression. It has been suggested that DNA-binding specificities are different among various NFκB dimers, which is linked to dimer-specific roles in gene regulation [41]. It was reported by a previous study that one c-Rel subunit was able to bind to a nonconsensus half-site of the DNA-binding domain with the other subunit anchoring at the consensus half-site [40]. Therefore, it is possible that a non-canonical NFκB binding site is located in p*USP16*-N5, which was regulated by other NFκB family members except p65. As LPS and TNFα are strong activators of NFκB pathway, we tried stimulate *USP16* gene by adding LPS and TNFα to cell culture medium and confirmed that both LPS and TNFα stimuli enhanced *USP16* transcription. By knocking out p65 in MEF cells, the effects of TNFα treatement on upregulating*USP16* gene expression was abolished.

DS has been associated with early onset and higher incidence of aging-related diseases such as AD [5, 42, 43]. DS patients develop early-onset AD (EOAD). Full trisomy of chromosome 21 inevitably causes the development of two pathological characteristics in AD brains, amyloid plaques and neurofibrillary tangles (NFTs). And by the age of 60, approximately two-thirds of individuals with DS suffer from dementia [44, 45]. The mechanisms linking DS to AD remain to be defined. It has been shown that the aging process is related with an impaired or exhausted ability of stem cells to renew themselves. *USP16*, in this case, may play a role in AD-related pathogenesis in DS. A number of genes on the chromosome 21 plays an important role in the AD pathogenesis. Duplication of amyloid β precursor protein (APP) gene was reported to cause autosomal dominant early-onset Alzheimer disease in five families [46], and various mutations in the APP gene has been identified in AD patients [47]. Our lab recently discovered that BACE2, another the chromosome 21-located gene, was a conditional BACE1 to facilitate AD pathogenesis [48]. NFκB signaling has been implicated in the AD pathogenesis. BACE1 cleaves APP to generate amyloid β protein (Aβ), a central component of neuritic plaques in the AD brains. Previously we found that NFκB p65 expression resulted in increased BACE1 promoter activity and BACE1 transcription [23]. We also demonstrated that non-steroidal anti-inflammatory drugs (NSAIDs) and inhibition of GSK3 signaling inhibited BACE1 transcriptional activation [23, 24]. Regulator of Calcineurin 1 (RCAN1*)* gene, a gene on Chromosome 21, has been implicated in pathogenesis of DS and AD [49]. We showed that RCAN1 expression was elevated in DS and AD, and its overexpression in primary neurons induced caspase-3 dependent apoptosis [3, 7, 8]. We demonstrated that the RCAN1 isoform 4 gene transcription was activated by NFκB signaling [50]. As we have demonstrated that NFκB promotes *USP16* gene transcription in this study, we attempt to explore the role of *USP16* in AD pathogenesis in future studies.

Although we have identified that *USP16* gene transcription was positively affected by NFκB, we also found three negatively regulatory elements in the *USP16* promotor region (+ 427 bp~ + 468 bp, + 130 bp~ + 150 bp, − 200 bp~ − 99 bp). These three negatively regulatory regions contain a common transcription factor binding site for YY1 (data not shown). The ubiquitous transcription factor YY1 is known to be a multifunctional protein that can either activate or repress gene expression depending upon the cellular context. Ying Yang 1 has been reported to play fundamental roles in embryogenesis, differentiation, replication, and cellular proliferation [51–54]. Ying Yang 1 involved in nervous system development, neuronal differentiation and function [55]. Further studies about whether YY1 could regulate *USP16* gene transcription and the potential impacts of *USP16* on DS pathogenesis may be warranted.

In conclusion, our study demonstrates that that *USP16* gene expression is tightly regulated at transcription level. NFκB signaling regulates the human *USP16* gene expression through three *cis*-acting elements. The results provide novel insights into a potential role of dysregulation of *USP16* expression in Alzheimer’s dementia in Down Syndrome.

## Supplementary information

**Additional file 1.** Supplementary Information.

## Data Availability

Described in Results and Methods section and the authors agree the availability upon request.

## Abbreviations

AD: Alzheimer’s disease
DS: Down syndrome
NFκB: Nuclear factor kappa-light-chain-enhancer of activated B cells
*USP16*: Ubiquitin specific peptidase 16
