# Supplementary material for: Transcriptional activation of USP16 gene expression by NFκB signaling
Source: Mol Brain. 2019 Dec 30;12:120. doi: 10.1186/s13041-019-0535-3 (PMC6937840; doi:10.1186/s13041-019-0535-3)
Supplement: Supplementary file 1 — Additional file 1. Supplementary Information. [file 13041_2019_535_MOESM1_ESM.docx]

**Supplementary Information**

**Primers for cloning promoter and deletion fragments in Figure 2**

-1856xhoIF: 5'-CCGCTCGAGCTGGAGCCTATGCCCTAC-3’

-1325XhoIF: 5'-CCGCTCGAGTCTCAGCAGTAAAAAGCAAG-3’

-653XhoIF: 5'CCGCTCGAGTATCTGACACCGTCTTTCG-3’

-200XhoIF: 5'CCGCTCGAGGAAACTCCAAGGCTCAGAC-3’

-99XhoIF: 5'CCGCTCGAGCCCCTAAACCCGAACATC-3’

-52XhoIF: 5'CCGCTCGAGACTAGCGTCAGAGCCGATG-3’

+1XhoIF: 5'CCGCTCGAGTAGCCACTTCCCATAATGC-3’

+21HindIIIR: 5’-TAC AAGCTTCGGCATTATGGGAAGTGG-3’

+40HindIIIR: 5'TAC AAGCTTAGCAATAACTTCCGGAAC-3’

+98HindIIIR: 5'TAC AAGCTTTCTTCCTCCTGGTGACG-3’

+110HindIIIR: 5'TAC AAGCTTCAGCCAGCTCCGTCTTC-3’

+130HindIIIR: 5'TAC AAGCTTTCATGGGCCTTTGGGC-3’

+150HindIIIR: 5'TAC AAGCTTGAGCCCATAACTGCATC-3’

+261HindIIIR: 5'TAC AAGCTTGCCTTCAAGAAGAGAACG-3’

+385HindIIIR: 5'TAC AAGCTTTCCACGGGGTCGAGAAG-3’

+427HindIIIR: 5'TACAAGCTTCTCCCCTTCTAACTGCTC-3’

+468HindIIIR: 5'TAC AAGCTTCACCCACCCATTTGCTCG-3’

**Primers used in Figure 3**

-393xhoIF: 5’- CCGCTCGAGGAGGGATATTCCGTCTC-3’

-512xhoIF: 5’- CCGCTCGAGTGGTGAAACCCCGTCTC-3’

-830xhoIF: 5’- CCGCTCGAGGACGGGATTTCACCACG-3’

**Oligonucleotide sequences in EMSA assay in Figure 4**

**wildtype NFκB consensus oligonucleotides**

Forward: AGTTGAGGGGACTTTCCCAGGC

Reverse: GCCTGGGAAAGTCCCCTCAACT

**mutant NFκB consensus oligonucleotides**

Forward: AGTTGAGGCCACTTTCCCAGGC

Reverse: GCCTGGGAAAGTGGCCTCAACT

**USP16-NFκB1 oligonucleotides**

Forward: TTATGGTGAATTACCTGTTTAC

Reverse: GTAAACAGGTAATTCACCATAA

**mutant USP16-NFκB1 oligonucleotides**

Forward: TTATGCTGAATTAAATGTTTAC

Reverse: GTAAACATTTAATTCAGCATAA

**USP16-NFκB2 oligonucleotides**

Forward: TAGAGACGGGATTTCACCACGT

Reverse: ACGTGGTGAAATCCCGTCTCTA

**mutant USP16-NFκB2 oligonucleotides**

Forward: TAGAGACGCCATTTCACCACGT

Reverse: ACGTGGTGAAATGGCGTCTCTA

**USP16-NFκB3 oligonucleotides**

Forward: CAATTGGTGAAACCCCGTCTCT

Reverse: AGAGACGGGGTTTCACCAATTG

**mutant USP16-NFκB3 oligonucleotides**

Forward: CAATTGGTCAAATTTCGTCTCT

Reverse: AGAGACGAAATTTGACCAATTG

**USP16-NFκB4 oligonucleotides**

Forward: GACAGAGGGATATTCCGTCTCA

Reverse: TGAGACGGAATATCCCTCTGTC

**mutant USP16-NFκB4 oligonucleotides**

Forward: GACAGAGCCATATTCCGTCTCA

Reverse: TGAGACGGAATATGGCTCTGTC

**USP16-3×NFκB oligonucleotides**

Forward: ACGGGATTTCACCACTTGGTGAAACCCCGTGAGGGATATTCCGTC

Reverse: GACGGAATATCCCTCACGGGGTTTCACCAAGTGGTGAAATCCCGT

**Primers for USP16 mRNA qPCR in Figure 5**

USP16 CDS+54F: 5'-GCTTTAGTGAATGTGGAATG-3'

USP16 CDS+235R: 5'-CTTGGCGTCAGATAGTGC-3'

GAPDH-F: CTCTGCTCCTCCTGTTCGAC

GAPDH-R: GCGCCCAATACGACCAAATC
